# 3D Printed Customized Facemask for Maxillary Protraction in the Early Treatment of a Class III Malocclusion: Proof-of-Concept Clinical Case

**DOI:** 10.3390/ma15113747

**Published:** 2022-05-24

**Authors:** Lorenzo Franchi, Alessandro Vichi, Patrizia Marti, Flavio Lampus, Simone Guercio, Annamaria Recupero, Veronica Giuntini, Cecilia Goracci

**Affiliations:** 1 Department of Experimental and Clinical Medicine, University of Florence, 50127 Florence, Italy; lorenzo.franchi@unifi.it (L.F.); veronica.giuntini@unifi.it (V.G.); 2 Dental Academy, University of Portsmouth, Portsmouth PO1 2QG, UK; alessandro.vichi@port.ac.uk; 3 Santa Chiara Fab Lab, Department of Social Political and Cognitive Sciences, University of Siena, 53100 Siena, Italy; patrizia.marti@unisi.it (P.M.); lampus.flavio@gmail.com (F.L.); s.guercio@gmail.com (S.G.); annamaria.recupero@gmail.com (A.R.); 4 Department of Medical Biotechnologies, University of Siena, 53100 Siena, Italy

**Keywords:** maxillary protraction facemask, face scanning, 3D printing, customization, Class III malocclusion

## Abstract

In order to improve fit and comfort, a maxillary protraction facemask customized to the patient’s anatomy was produced by means of 3D face scanning, digital design and additive manufacturing. An 8-year-old patient in need of early treatment for the Class III malocclusion received a rapid palatal expander and a Petit-type facemask, whose components were digitally designed on a 3D scan of the patient’s face. For face scanning, the iPad Pro 2018 tablet (Apple, Cupertino, CA, USA) with the Bellus3D DentalPro application (Bellus3D, Campbell, CA, USA) was used. Facemask components were modelled with 3D Blender software. The rests were 3D printed in BioMed Clear biocompatible resin (Formlabs, Somerville, MA, USA), and the bar in stainless steel. For greater comfort, the internal surface of the rests was lined with a polymer gel pad (Silipos, Niagara Falls, NY, USA). The manufacturing procedure of the customized facemask is patented. The patient wore the facemask at night for a period of 9 months. The patient’s experience was evaluated with a questionnaire at 1 week, 3, 6, and 10 months of treatment. The customized facemask was well accepted by the patient and obtained the expected treatment outcome. Furthermore, 3D face scanning, 3D modelling and 3D printing allow for the manufacturing of customized facemasks with improved fit and comfort, favoring patient compliance and treatment success.

## 1. Introduction

The correction of Class III malocclusions still represents a challenge for the orthodontist [1]. Addressing the Class III malocclusion at a growth stage when the circum-maxillary sutures are still immature [2,3,4,5], usually before age 10 [6], has been advised to maximize the orthopedic effect, for the benefit of an early improvement in the dento-skeletal relationship and facial esthetics, and with the intention to limit the need or the complexity of a future surgical correction [1,2].

Clinicians have resorted to a variety of appliances for the early treatment of Class III malocclusions [7]. Nevertheless, abundant good-quality evidence has been collected in support of the maxillary protraction facemask. Such an extraoral device, used most often in combination with a rapid palatal expander as the tooth-borne or bone-borne intraoral anchorage, has proved effective at promoting forward growth of the maxilla and limiting mandibular growth [1,2,6,7,8,9,10].

It is also evident that the outcome of facemask treatment is strongly influenced by the patient’s compliance with wearing the appliance for the requested time. Wearing times, ranging between 14 h a day and full time, have been prescribed by different practitioners [11]. However, patients’ experience with the currently marketed facemasks, available only in standard shapes and in a minimal choice of sizes, has often been dissatisfactory [11,12,13,14]. Among several intra- and extraoral orthodontic appliances assessed for acceptability and attractiveness, facemasks had the worst patient rating [13]. Typical complaints regard facemask’s bulkiness and instability, while the most common undesirable effect is the occurrence of skin irritations, particularly at the chin level, due to the pressure applied by the pads [15]. Sores can be produced at the mouth corners or on the lower lip by impinging elastics [15]. Additionally, gingival recessions of the lower incisors have been observed due to the pressure applied by a chin cup accidentally dislodged toward the gingival margin.

Facemask fit and comfort would certainly be improved if the shape and size of the facemask components could be customized to the patient’s anatomy.

For this purpose, Cacciatore et al. [16] proposed to reline the acrylic resin chin cup of the standard facemasks with a putty-consistency polyvinyl siloxane, whereas Ierardo et al. [14] introduced underneath the chin pad, a 3-mm thick layer of soft-bite material thermoformed on a plaster model of the patient’s chin.

Earlier attempts to fully customize the facemask have involved recording a plaster [17] or alginate [18] impression of the patient’s face, which is not a pleasing procedure for a child.

Nowadays, a three-dimensional (3D) image of the patient’s facial anatomy can be acquired in a totally non-invasive way by means of a facial scanner. The facemask components can be custom designed on the 3D face scan with a 3D modelling software, and the modelled parts can be additively manufactured by means of a 3D printer.

By utilizing these innovative technologies, a maxillary protraction facemask of the Petit-type with rests and a midline bar customized to the patient’s facial anatomy was produced and clinically utilized in a child in need of early treatment for Class III malocclusion. This case report describes the procedures followed to manufacture the customized facemask and presents the clinical outcome of treatment.

## 2. Materials and Methods

**Figure 1:**
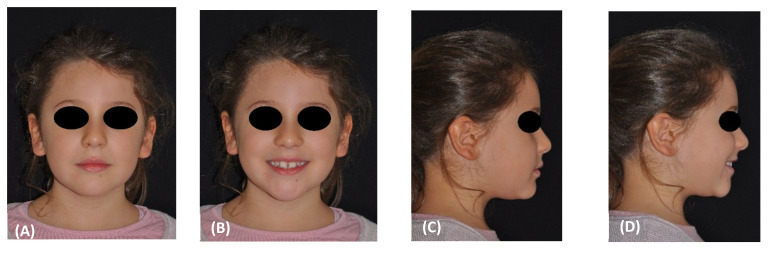

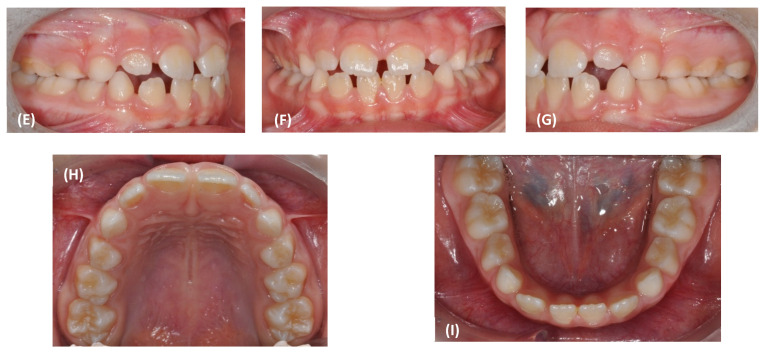
(**A**–**D**) Pretreatment facial photographs. (**E**–**G**) Pretreatment intraoral frontal and lateral photographs. (**H**,**I**) Pretreatment occlusal upper and lower arch photographs.

The patient was an 8-year-old female, who was referred to the Orthodontic Clinic of the Careggi University Hospital in Florence for an orthodontic evaluation. In the frontal view, the patient exhibited facial symmetry and well-proportioned face thirds in the vertical plane (Figure 1A,B). The profile appeared flat (Figure 1C,D).

Intraorally, she presented with a mild Class III dental relationship in the mixed dentition and minimal overjet, despite the proclination of the upper incisors as a dental compensation (Figure 1E–G).

A constriction of the maxillary arch was also evident from the occlusal photograph (Figure 1H,I).

**Figure 2:**
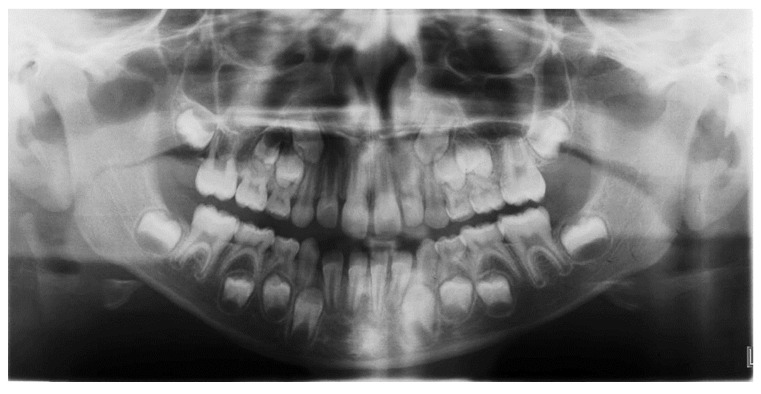
Pretreatment panoramic radiograph.

The panoramic radiograph revealed a discrepancy between the space available and the space requested for the eruption of the permanent teeth, particularly in the canine area (Figure 2).

**Figure 3:**
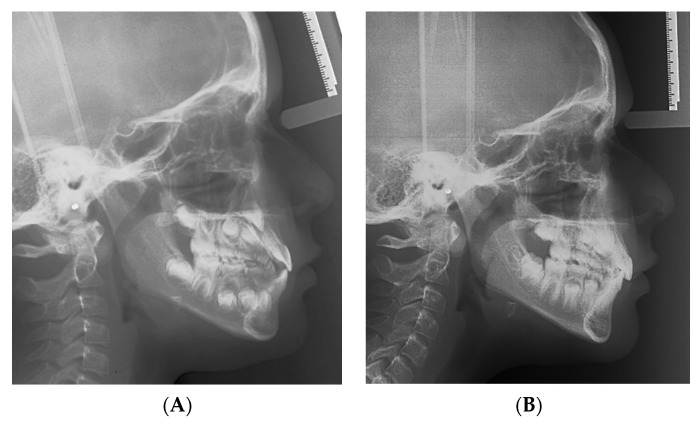
Pretreatment (**A**) and posttreatment (**B**) lateral cephalogram.

A Class III maxillo-mandibular sagittal relationship emerged from the cephalometric analysis of the pretreatment lateral cephalogram (Figure 3A), while the vertical skeletal relationships appeared normal (Table 1). The cervical vertebral maturation stage was CS1.

**Figure 4:**
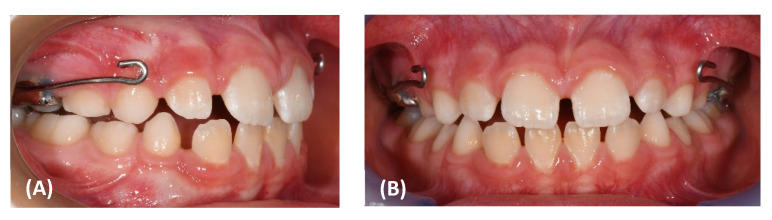

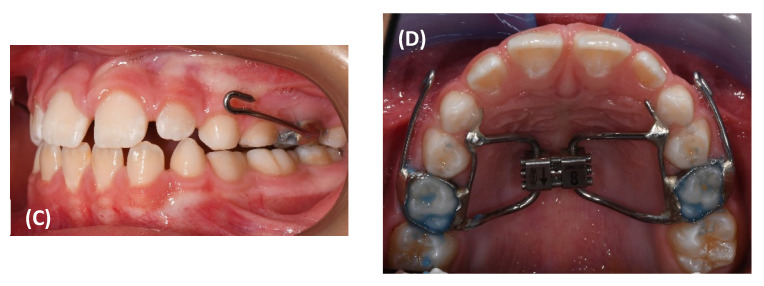
(**A**–**D**) Intraoral photograph of the Hyrax-type rapid palatal expander in place.

The treatment plan involved the application of a Hyrax-type rapid palatal expander (RPE) (Figure 4A–E) to relieve crowding and disrupt the maxillary sutures, making the maxilla more responsive to the protracting force applied by a facemask. The expansion screw was activated at the rate of a one-quarter turn per day, corresponding to 0.2 mm of expansion. The active expansion was stopped when the lingual cusp of the upper second deciduous molars approximated the buccal cusps of the lower second deciduous molars.

The decision was made to manufacture a Petit-type facemask, with rests and a midline bar customized to the patient’s facial anatomy.

**Figure 5:**
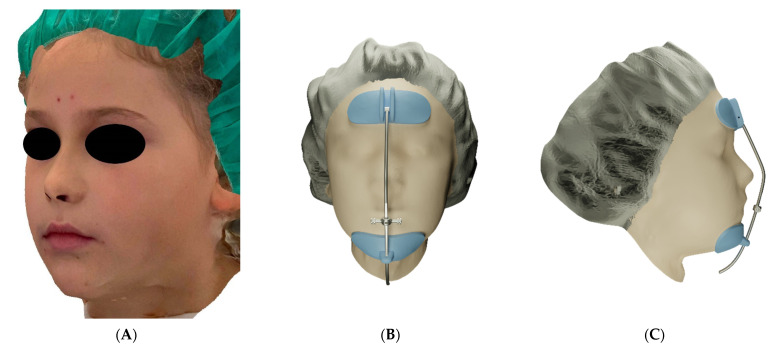
(**A**) The 3D scan of the patient’s face. (**B**,**C**) The 3D model of the customized facemask.

The iPad Pro 2018 tablet (Apple Inc., Cupertino, CA, USA) was used with the Bellus3D DentalPro application (Bellus3D, Campbell, CA, USA) to perform a 3D scan of the patient’s face. Scanning was done in a bright room and care was taken to keep the patient’s hair away from the forehead and to avoid the projection of shadows on the face (Figure 5A).

The patient was asked to sit in a resting posture and to keep the teeth in occlusion and the lips relaxed. A robotic voice guided the child to tilt and rotate the head as needed for proper image capturing, which was completed within a few seconds. The acquired 3D image was then exported in .stl format. The frontal support, the chin rest and the central bar of the facemask were modelled on the 3D image of the patient’s face, using the 3D modelling software, Blender (https://www.blender.org/, accessed on 27 February 2022) (Figure 5B,C) [19].

**Figure 6:**
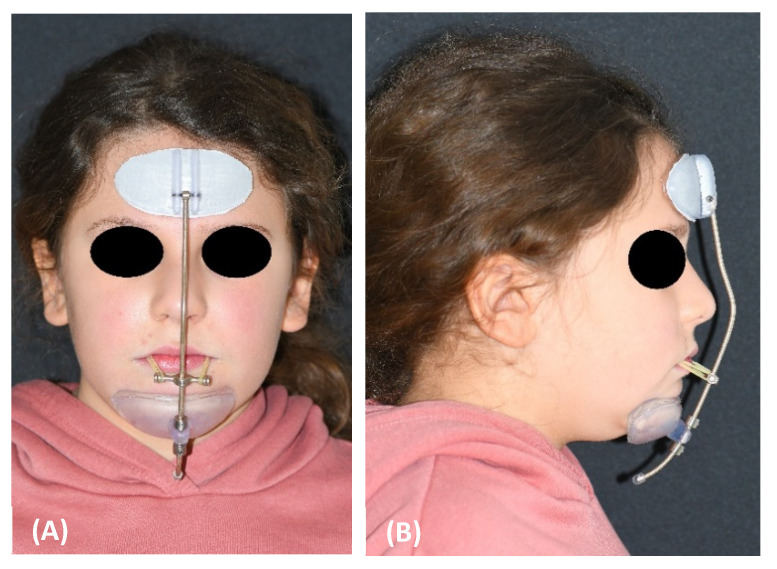
(**A**,**B**) The customized facemask in place.

Then, the rests were printed with BioMed Clear biocompatible resin using the Form 3 3D printer (Formlabs, Somerville, MA, USA), while the bar was printed in stainless steel [20]. After the necessary finishing of the printing material, the various components were assembled to complete the customized facemask. For greater comfort, the internal surface of the front and chin supports was coated with protective pads in polymeric gel with a moisturizing and soothing action for the skin, already on the market for applications in orthopedics and dermatology (Silipos, Niagara Falls, NY, USA). The printing, finishing and assembly of the individualized components, as well as the coating of the internal surface of the supports, were performed at the Santa Chiara Fab Lab digital manufacturing laboratory of the University of Siena. The customized device was then delivered to the patient by the orthodontist, who also established the vertical positioning of the horizontal support, which could slide along the bar so that the elastics would have a 30° downward inclination relative to the occlusal plane. Additionally, the orthodontist provided the parents with the necessary instructions regarding how to fix the elastic bands to the rapid intraoral expander and the recommended application times. The manufacturing procedure of the customized facemask is patented (European Patent n. EP 3752091, US Patent n. US20200397536). The study was conducted in accordance with the Declaration of Helsinki and approved by the Regional Pediatric Ethics Committee (CEP 236/2020). Informed consent was obtained from the patient’s parents. The facemask was delivered to the patient at the completion of the RPE phase. Maxillary protraction was performed by applying an elastic force of 500 g per side (Figure 6).

The patient was advised to wear the facemask for 14 h a day. The patient’s experience with the customized mask was evaluated through a questionnaire that the patient, with the help of her parents, filled out after 1 week, and after 3, 6, and 10 months since appliance delivery (Supplementary Materials). The questionnaire included a visual analogue scale (VAS) for pain, ranging from 0 (no pain) to 10 (worst imaginable pain). The questionnaire is also meant to assess the patient’s compliance with facemask therapy, as well as the occurrence of any complications, particularly skin irritations. The patient’s satisfaction with the therapy was also measured with a VAS scale, in which 0 indicated ‘no satisfaction’ and 10 ‘maximum satisfaction’.

## 3. Results

**Figure 7:**
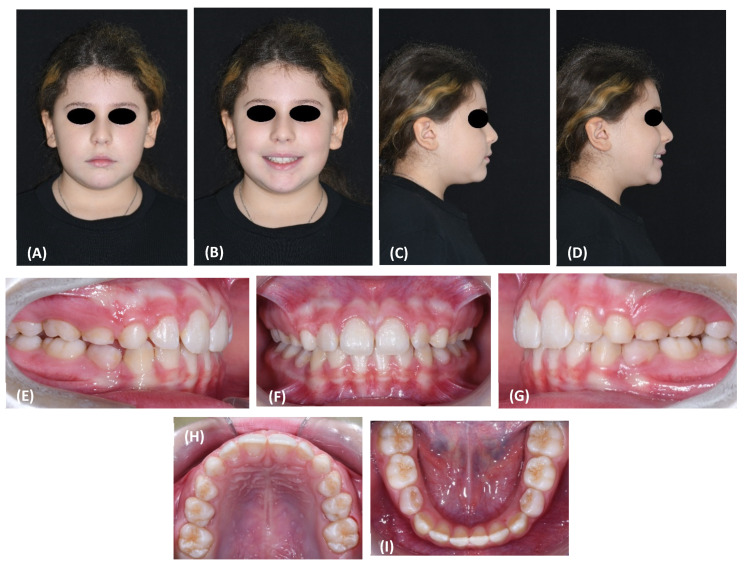
(**A**–**D**) Posttreatment facial photographs. (**E**–**G**) Posttreatment intraoral frontal and lateral photographs. (**H**,**I**) Posttreatment occlusal upper and lower arch photographs.

The customized facemask showed an excellent adaptation to the anatomy of the face and was lighter than the standard facemask. According to the patient and her parents, the facemask was worn for a daily average of 9 h, mostly at night. The experience with the facemask was positively evaluated by the patient. At the 3-, 6-, and 10-month evaluation, the patient then rated it as a 7.5 for her satisfaction with the treatment on a 1–10 visual analogue scale. After 10 months, the facemask treatment was stopped, the RPE was removed, and the patient was given a removable mandibular retractor [21] to be worn at night for 12–18 months in the interim period before the second phase of treatment, if needed. At the completion of the 10-month facemask therapy, the patient exhibited very pleasant face and profile esthetics (Figure 7A–D).

Intraorally, a cusp-to cusp molar and deciduous canine relationship was present and a positive overjet and overbite (2.7 and 3.6 mm, respectively) were achieved (Figure 7E,G).

The posttreatment cephalogram (Figure 3B, Table 1) showed that the ANB angle increased by 3.7 degrees, while the Wits appraisal was reduced by 3.2 mm. Both the inclinations of the palatal plane and of the mandibular plane to the cranial base (S-N) remained unchanged. The mandibular angle Co-Go-Me was reduced by 6.1 degrees. The inclination of the upper incisor to the palatal plane decreased by 5.4 degrees, while the inclination of the lower incisors to the mandibular plane increased by 4.3 degrees.

## 4. Discussion

Previous clinical experience with the protraction facemasks available on the market has revealed several limitations of such standard devices, due to their inability to adapt well to all patients [14] especially children of a very young age, exhibiting particularly small faces or children affected by craniofacial deformities that often present with a Class III malocclusion. It is a common occurrence for orthodontists to collect patients’ and parents’ complaints about the device being cumbersome, unstable, and, overall, uncomfortable. Poor adaptation and scarce patient acceptance of the facemask can compromise the success of treatment. Nevertheless, the introduction of current technologies for 3D imaging, 3D modelling, and 3D printing represents a breakthrough in the custom production of these devices, which could even be implemented by a dental laboratory.

**Figure 8:**
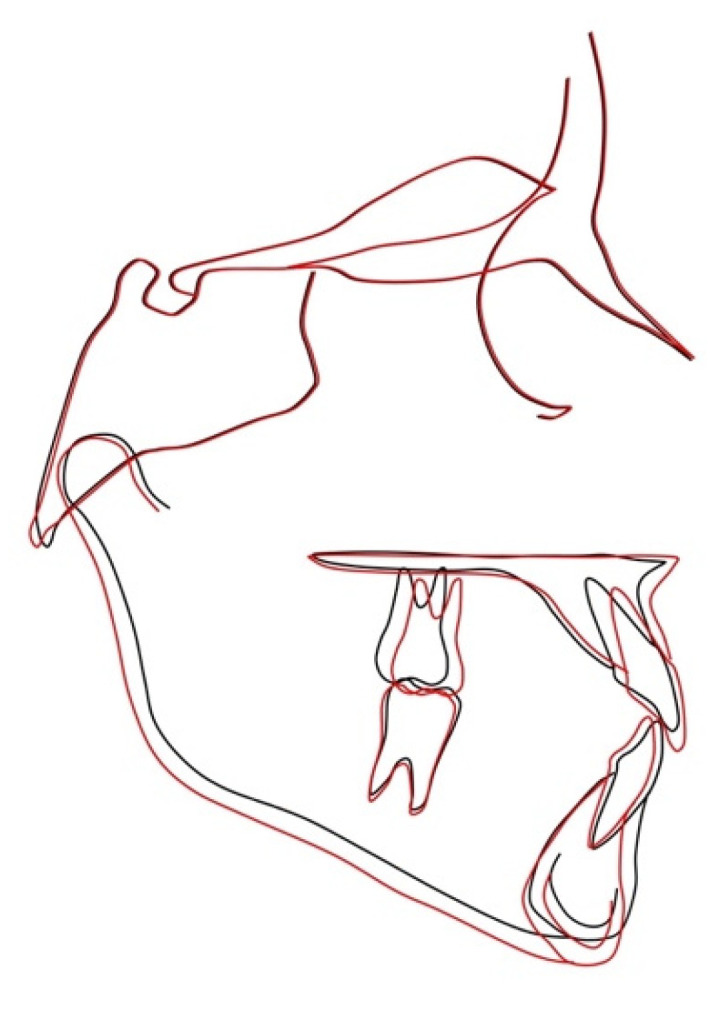
Superimposition of pretreatment (black) and posttreatment (red) cephalometric tracings on the stable basicranial structures.

This case report indeed demonstrated that the 3D printed facemask customized to the patient’s anatomy could be successfully manufactured, was well received by the patient, and obtained the expected treatment outcome. Following RPE and facemask therapy, the patient exhibited a correct inter-arch relationship on the transversal, sagittal, and vertical planes (Figure 7). Moreover, at the skeletal level, the antero-posterior jaw relationship was improved, with a remarkable increase in overjet and overbite and without any negative effect on divergence (Figure 8, Table 1). The positive finding of a good efficacy of the therapy was complemented by the reassuring observation that the appliance was well tolerated by the patient throughout the 9 months of application.

Moreover, the use of innovative technologies in the effort to increase the appliance comfort was well appreciated by the patient’s parents.

The customized protraction facemask that was tested in this clinical case actually presents as a ‘sustainable’ device on both the patient’s and the orthodontist’s sides.

The face scan was indeed acquired in a few seconds and in a non-invasive way by means of an iPad Pro, which is a portable, relatively inexpensive, and easily purchasable device. Since the TrueDepth camera system used by the Bellus3D Dental Pro application for face scanning is built into any Apple device running IOS 12.2 or later, even an iPhone X would be adequate for the purpose.

Recent research has indicated that the Bellus3D application compared well with more expensive devices for face scanning in terms of accuracy [22,23,24,25]. Although the Bellus3D application has been made unavailable for download since 1 December 2021, several other systems are currently available for 3D face scanning [22,23,24,25].

Scans were exported in standard tessellation/triangulation language (stl), which is an open file format compatible with several softwares for digital design and is supported by most 3D printers. A scenario can be figured out in the near future, for when orthodontists from all over the world could simply email the .stl file of the patient’s face scan to a laboratory, and, within a few days, receive the 3D printed customized facemask ready for delivery.

Further developments of the project involve the use of additional digital fabrication tools to create a gallery of decorations the patient can choose from, in order to further individualize the facemask and increase the patient’s acceptance of the device. Even more importantly, in the digital manufacturing process, the placement within the facemask of sensors to monitor wear time can be implemented [12,14]. A clinical trial on the use of sensorized custom-made facemasks in the early treatment of Class III malocclusions is currently ongoing at the Orthodontic Clinic of the Careggi University Hospital in Florence. Wear time monitoring allows for objectively assessing to what extent a patient’s compliance influences the outcome of facemask therapy and whether there is a threshold value of wear time for the treatment to be clinically effective [26,27].

To additively manufacture the customized frontal and mental pad, a photopolymer was chosen that, according to the manufacturer, is indicated for applications requiring long-term skin or mucosal membrane contact [28]. BioMed Clear Resin has indeed been USP Class VI certified according to ISO 10993-5, -10 and ISO 13485.26.

To reduce the risk for skin irritation, a commonly encountered problem during facemask treatment, the forehead and chin pads were interiorly lined with a gel cushion that, according to the manufacturer, protects the skin from pressure or friction, and gradually diffuses USP medical grade mineral oil, having a hydrating action [29]. Nevertheless, the customized pads were so well-fitting that the pressure applied by the protracting elastics can be expected to be uniform on the contacting skin.

**Figure 9:**
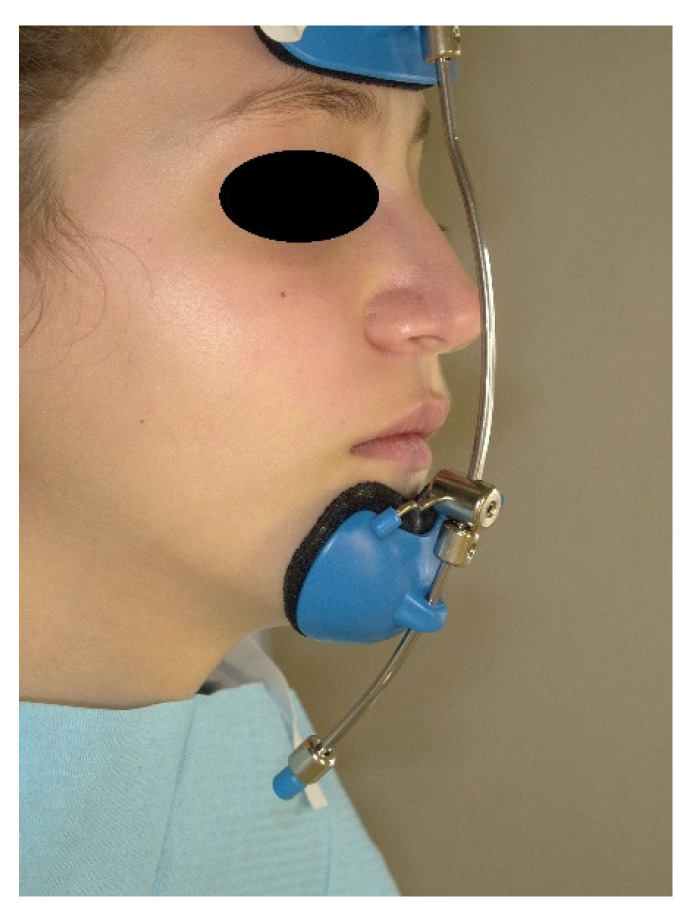
Oversized midline bar in a marketed standard facemask.

Moreover, the customization of the midline bar represents a great advancement, as the bar of customized facemasks results as oversized in many patients and often needs to be shortened, particularly at the bottom end, where a too-long bar can hit and hurt the neck or chest when the patient tilts the head down (Figure 9).

In the presented case, it was decided to print the midline and the crossbar in stainless steel in order for these structures to have the strength needed to resist the protraction forces without deformation. However, in consideration of the recent introduction of several fiber-reinforced or nanofilled 3D printable polymers [30,31], it can be predicted that these innovative materials with enhanced mechanical properties can soon replace the metal in the manufacture of the facemask bars. This would result in a reduction in the facemask weight which would further increase comfort. It would streamline the workflow and perhaps allow for a cost reduction, also considering the recent rise in the price of metals.

The cost estimation for the customized facemask is about 100.00 euros, which is a bit higher compared to the price of the commercially available facemask (about 70.00 euros).

The described case is part of a larger clinical investigation on customized facemasks that is being undertaken at the University of Florence, with the objective of advancing the device from the prototype level demonstrated in the laboratory (technology readiness, level 4) to validation with a clinical trial (technology readiness, level 7).

## 5. Conclusions

The presented case provided a proof-of-concept for the manufacturing and effective clinical management of a 3D printed protraction facemask whose components were customized to the patient’s anatomy, thus improving the fit and comfort of the device as compared to the standard facemasks in the market. This advancement was attained by applying the innovative technologies of face scanning as a non-invasive method for 3D image acquisition, digital design and additive manufacturing. The customized facemask was well received by the patient and produced the expected treatment results. The successful clinical outcome of this facemask prototype prompts further developments of the idea, encompassing the introduction of sensors for treatment monitoring and the use of novel 3D printable polymers with enhanced mechanical behavior, that may advantageously replace the metal, in the pursuit of a greater ‘sustainability’ of the therapy for the patient and for the orthodontist as well.

## 6. Patents

The manufacturing procedure of the customized facemask is patented (European Pa-tent n. EP 3752091, US Patent n. US20200397536).

## Figures and Tables

**Table 1:** Cephalometric analysis.

Variables	Normal Values	Pretreatment	Posttreatment
Angular degrees			
SNA	82	87.7	89.6
SNB	80	85.3	83.5
ANB	2	2.5	6.2
SN to Palatal Plane	8	9.6	9.6
SN to Mand. Plane	32	33.3	33.5
Co-Go-Me	125	136.1	130
Upper Inc. to Palatal Pl	110	119	113.6
Lower Inc. to Mand. Pl.	90	87.5	91.8
Linear, mm			
Wits	0	−6.3	−3.1
Overjet	2.5	0.4	2.7
Overbite	2.5	1.1	3.6

## Data Availability

Not applicable.

## Supplementary Materials

The following supporting information can be downloaded at: https://www.mdpi.com/article/10.3390/ma15113747/s1.
