# Supplementary material for: 3D Printed Customized Facemask for Maxillary Protraction in the Early Treatment of a Class III Malocclusion: Proof-of-Concept Clinical Case
Source: Materials (Basel). 2022 May 24;15(11):3747. doi: 10.3390/ma15113747 (PMC9181288; doi:10.3390/ma15113747)
Supplement: Supplementary file 1 [file materials-15-03747-s001.zip › materials-1713547-Supplementary Materials.pdf]

materials

MDPI

Supplementary Materials

3D Printed Customized Facemask for Maxillary Protraction in the Early Treatment of a Class III Malocclusion: Proof-of-Concept Clinical Case

Lorenzo Franchi [1], Alessandro Vichi [2], Patrizia Marti [3], Flavio Lampus [3], Simone Guercio [3], Annamaria Recupero [3], Veronica Giuntini [1] and Cecilia Goracci [4,*]

[1] Department of Experimental and Clinical Medicine, University of Florence, 50127 Florence, Italy; lorenzo.franchi@unifi.it (L.F.); veronica.giuntini@unifi.it (V.G.)
[2] Dental Academy, University of Portsmouth, Portsmouth PO1 2QG, UK; alessandro.vichi@port.ac.uk
[3] Santa Chiara Fab Lab, Department of Social Political and Cognitive Sciences, University of Siena, 53100 Siena, Italy; patrizia.marti@unisi.it (P.M.); lampus.flavio@gmail.com (F.L.); s.guercio@gmail.com (S.G.); annamaria.recupero@gmail.com (A.R.)
[4] Department of Medical Biotechnologies, University of Siena, 53100 Siena, Italy
* Correspondence: cecilia.goracci@unisi.it; Tel.: +39-0577-585771

Patient Questionnaire

Please complete this questionnaire together with your child 4 times, once after one week, once after 3 months, once after 6 months, and once after 10 months (at the end of treatment) and bring it to each subsequent dental visit.

Questionnaire to be completed after the first week

Pain scale

Please draw a mark with a pen to indicate the amount of pain that you experienced. For example, if high pain was found in the first week you may draw an X with the pen at the score 8 or 9.

0-------1-------2-------3-------4-------5-------6-------7-------8-------9-------10

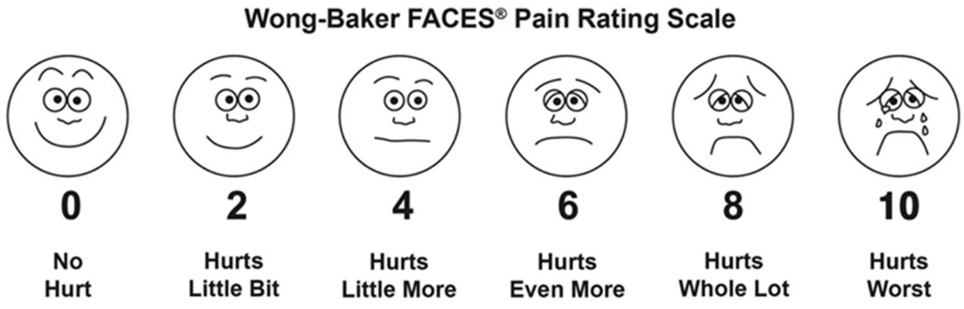

Please indicate the area where you felt pain________________________________

Degree of compliance

How many hours per day was the facemask worn? Report an average number of hours:_______

Do you wear the facemask every day without being reminded by your parents?
Yes No

Do you often ask to be allowed to remove the facemask? Yes No

When you don't want to wear the facemask my parents:
explain to me that it is important for my teeth Yes No
they offer me a small reward (e.g., play a game I like) Yes No
Problems with the facemask
Have you had skin irritation (reddened skin) on your forehead? Yes No
Have you had skin irritation (reddened skin) on your chin? Yes No
Have you had irritation at the corners of your mouth? Yes No
Have you had irritation on the lower lip? Yes No
Did you sleep badly with the facemask on? Yes No
Have you had any other discomfort due to the facemask? Yes No
If yes, which ones? ____
____

Questionnaire to be completed after 3 months

Pain scale

Please draw a mark with a pen to indicate the amount of pain that you experienced. For example, if high pain was found in the first week you may draw an X with the pen at the score 8 or 9.

0-------1-------2-------3-------4-------5-------6-------7-------8-------9-------10

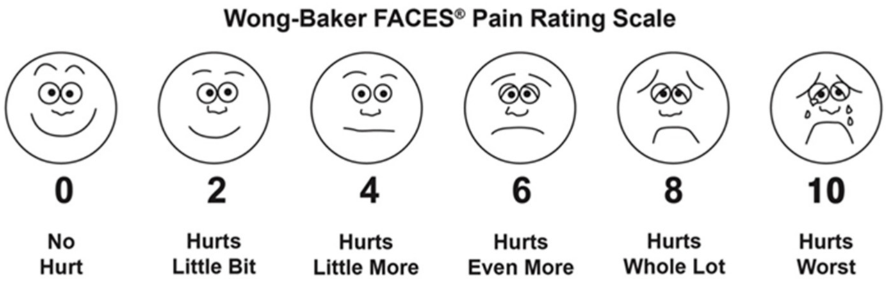

Please indicate the area where you felt pain______________________________

Patient satisfaction scale

0-------1-------2-------3-------4-------5-------6-------7-------8-------9-------10
No satisfaction with therapy — Greater imaginable satisfaction with therapy

Degree of compliance

How many hours per day was the facemask worn? Report an average number of hours: _______

Do you wear the facemask every day without being reminded by your parents? Yes No

Do you often ask to be allowed to remove the facemask? Yes No
When you don't want to wear the facemask my parents:
explain to me that it is important for my teeth Yes No
they offer me a small reward (e.g., play a game I like) Yes No
Problems with the facemask
Have you had skin irritation (reddened skin) on your forehead? Yes No
Have you had skin irritation (reddened skin) on your chin? Yes No
Have you had irritation at the corners of your mouth? Yes No
Have you had irritation on the lower lip? Yes No
Did you sleep badly with the facemask on? Yes No
Have you had any other discomfort due to the facemask? Yes No

If yes, which ones?____
____

Questionnaire to be completed after 6 months

Pain scale

Please draw a mark with a pen to indicate the amount of pain that you experienced. For example, if high pain was found in the first week you may draw an X with the pen at the score 8 or 9.

0-------1-------2-------3-------4-------5-------6-------7-------8-------9-------10

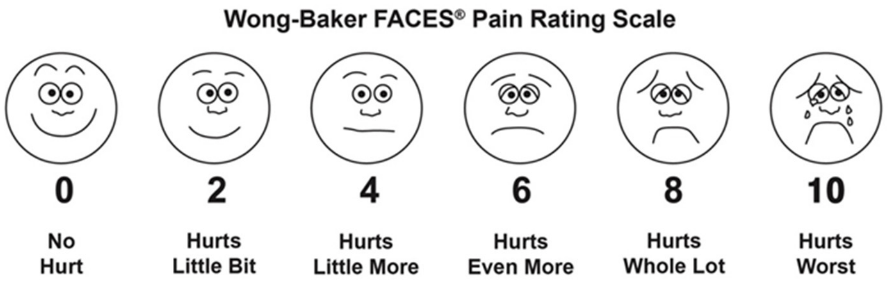

Please indicate the area where you felt pain______________________________

Patient satisfaction scale

0-------1-------2-------3-------4-------5-------6-------7-------8-------9-------10

No satisfaction with therapy — Greater imaginable satisfaction with therapy

Degree of compliance

How many hours per day was the facemask worn? Report an average number of hours:_______

Do you wear the facemask every day without being reminded by your parents?
Yes No

Do you often ask to be allowed to remove the facemask? Yes No

When you don't want to wear the facemask my parents:

explain to me that it is important for my teeth Yes No

they offer me a small reward (e.g., play a game I like) Yes No

Problems with the facemask

Have you had skin irritation (reddened skin) on your forehead? Yes No

Have you had skin irritation (reddened skin) on your chin? Yes No

Have you had irritation at the corners of your mouth? Yes No

Have you had irritation on the lower lip? Yes No

Did you sleep badly with the facemask on Yes No

Have you had any other discomfort due to the facemask? Yes No

If yes, which ones? ____
____

Questionnaire to be completed after 10 months (end of therapy)

Patient satisfaction scale

0-------1-------2-------3-------4-------5-------6-------7-------8-------9-------10

No satisfaction with therapy — Greater imaginable satisfaction with therapy

Degree of compliance

How many hours per day was the facemask worn? Report an average number of hours:________

Do you wear the facemask every day without being reminded by your parents? Yes No

Do you often ask to be allowed to remove the facemask? Yes No

When you don't want to wear the facemask my parents:

explain to me that it is important for my teeth Yes No

they offer me a small reward (e.g., play a game I like) Yes No

Problems with the facemask

Have you had skin irritation (reddened skin) on your forehead? Yes No

Have you had skin irritation (reddened skin) on your chin? Yes No

Have you had irritation at the corners of your mouth? Yes No

Have you had irritation on the lower lip? Yes No

Did you sleep badly with the facemask on? Yes No

Have you had any other discomfort due to the facemask? Yes No

If yes, which ones? ____

____

Complications

Patient name……………………………………………………………………….

Patient number ……………

Date…………………………

Describe the complication

………………………………………………………………………………………………
………………………………………………………………………………………………
………………………………………………………………………………………………
……………

Date of complication……………………Date of resolution……………………

Severe ○Yes ○No

NOTE: In case of a SEVERE complication please fill out this form and inform the principal investigator

Result ○Resolved without consequences ○Resolved with consequences ○Not resolved ○Death ○Unknown / Lost at follow-up

Treatment of the complication for this event ○No ○Yes (if yes, describe)

____

____

Relationship with the study ○No ○Possible ○Probable ○Proven

The complication resulted in hospitalization ○Yes ○No

Date of admission…………………… Data of discharge……………………
